# Mind wandering increases linearly with text difficulty

**DOI:** 10.1007/s00426-021-01483-9

**Published:** 2021-02-12

**Authors:** Rebecca Kahmann, Yesim Ozuer, Claire M. Zedelius, Erik Bijleveld

**Affiliations:** 1 Behavioural Science Institute, Radboud University, P.O. Box 9104, 6500HE Nijmegen, The Netherlands; 2 Psychological and Brain Sciences, University of California, Santa Barbara, CA USA

## Abstract

Although mind wandering during reading is known to be affected by text difficulty, the nature of this relationship is not yet fully understood. To examine this issue, we conducted an experiment in which participants read non-fiction texts that varied along five levels of difficulty under naturalistic conditions. Difficulty levels were determined based on Flesch–Kincaid Grade Levels and verified with Coh–Metrix indices. Mind wandering was measured with thought probes. We predicted that text difficulty and mind wandering have a U-shaped (i.e., quadratic) relationship. Contrary to our expectations, but in line with some prior studies, mind wandering linearly increased with text difficulty. Additionally, text interest moderated the effect of text difficulty on mind wandering. Finally, mind wandering was associated with worse performance on a comprehension test. Together, our findings extend previous work by showing that (a) a linear relationship between difficulty and mind wandering exists during common page-by-page reading of pre-existing texts and that (b) this relationship holds across a broad range of difficulty levels.

**Supplementary Information:**

The online version contains supplementary material available at 10.1007/s00426-021-01483-9.

## Introduction

When people’s *mind wanders* while reading, their mind is not fully processing the text; thus, mind wandering hinders text comprehension (Broadway et al. 2015; Feng et al. 2013; Smallwood et al. 2008; Soemer et al. 2019; Unsworth and McMillan 2013). As students (regardless of their level) and employees (regardless of their tasks) often learn through reading, mind wandering can harm people’s educational and occupational career (Kane and McVay 2012; Lindquist and McLean 2011; Smallwood and Schooler 2015). In this study, we examine how text difficulty affects mind wandering. We became interested in text difficulty as this parameter can readily be controlled, e.g., teachers may tailor the difficulty of teaching materials. As such, in the long run, we hope this research will contribute to the extent we can control mind wandering in applied settings.

## Text difficulty effects on mind wandering

*Mind wandering* refers to attention drifting away from the current task, towards task-unrelated or stimulus-independent mental content. Several prior experiments have studied the impact of text difficulty on mind wandering (Feng et al. 2013; Forrin et al. 2019; Fulmer et al. 2015; Giambra and Grodsky 1989; Mills et al. 2013, Mills et al. 2015; Soemer et al. 2019; Soemer and Schiefele 2019), but the issue is not yet resolved. On the one hand, research suggests that easy (compared to difficult) texts are more susceptive to mind wandering. For example, in one experiment (Fulmer et al. 2015), college students read educational texts that were tuned to be either easy or difficult to read. Students mind wandered more when reading easy texts, at least when they expected the texts to be uninteresting. This finding can be explained by the theory that mind wandering and reading compete for the allocation of cognitive control (Smallwood and Schooler 2006). As easy texts demand the allocation of less cognitive control for task-related processes, there should be greater opportunities to simultaneously process task-unrelated content. So, during easy (but not difficult) texts, the mind has the opportunity to wander. This view, sometimes called the *executive resource hypothesis*, is well-supported by studies outside the reading domain, e.g., by research that used working memory tasks (for a review, see Smallwood and Schooler 2015).

On the other hand, and in contrast to the research addressed above, several studies suggest that difficult (vs. easy) texts are more conductive to mind wandering (e.g., Feng et al. 2013; Mills et al. 2013, 2015; Soemer et al. 2019; Soemer and Schiefele 2019). To explain this finding, it helps to consider that readers need to build a *situational model* to be fully engaged in reading, and to comprehend a text. A situational model refers to an extensive mental representation of the meaning of the concepts and events described in the text, their implied context, and their connection to pre-existing knowledge (Feng et al. 2013; Smallwood 2011). The situational model also helps the reader to understand how different events explained in the text are connected to each other, which is crucial to understand a text as an entity. With increasing text difficulty, building a situational model is increasingly likely to fail. When this happens, people are thought to disengage from the text and mind wander.

Traditionally, mind wandering is assumed to be initiated mostly unintentionally (e.g., some theories conceptualize mind wandering as a by-product of attentional failure, Kane and McVay 2012). However, recent theories suggest that mind wandering can also happen intentionally, in that people voluntarily choose to disengage from their current task (Seli et al. 2016a, b). Research has shown that unintentional mind wandering tends to happen especially during difficult tasks, whereas intentional mind wandering tends to happen during easy tasks (Seli et al. 2016a, b). Although this dissociation seems to be clear-cut during cognitive tasks, such as the sustained attention to response task (SART), this dissociation does not clearly extend to reading. Specifically, one study showed that reading difficult texts was associated with more unintentional *and* more intentional mind wandering (Soemer and Schiefele 2019). Thus, the distinction between unintentional and intentional mind wandering cannot a priori explain the mixed findings in the reading domain. Nevertheless, theories on the role of intentionality in mind wandering may provide a useful starting point for explaining the mixed findings concerning text difficulty and mind wandering. Specifically, the insight that mind wandering may be voluntary suggests that a motivational approach to studying mind wandering may be viable (see also Seli et al. 2015; Seli et al. 2019; Soemer & Schiefele, 2019).

In the current study, we draw from the models of motivation–cognition interactions to make sense of the mixed findings concerning text difficulty and mind wandering (Shenhav et al. 2017). These models are based on the classic assumption that humans (and other organisms) avoid effort that is not proportional to the reward expected to result from it—in other words, people avoid effort that is not “worth it”. This behavioral principle has first been applied to behavioral effort (e.g., Hull 1943), and has since been extended to cognitive effort (e.g., Aridan et al. 2019; Dora et al. 2020; Kool et al. 2010). Phenomenologically, effort feels aversive (e.g., Dunn et al. 2016; Inzlicht et al. 2018; Saunders et al. 2017); however, exerting more effort is often associated with greater reward. Therefore, to decide whether to invest effort, people make cost–benefit analyses. When faced with a cognitively demanding task, people weigh its potential benefits (How rewarding will it be?) against its potential direct costs (How much control is needed for this task?) and opportunity costs (What else could I do instead?).

We suggest that such cost–benefit analyses can be applied to the context of mind wandering and reading. First, we assume that the more difficult a text is, the more cognitive effort is required to build a situational model. Hence, with increasing text difficulty, the potential benefits of reading become less and less likely to be worth the increasing cognitive costs, triggering people to process other mental content. Thus, we expect mind wandering to occur when people read (very) difficult texts. By contrast, for (very) easy texts, however, people need to allocate only little cognitive effort to reading. Thus, when people read easy texts, cognitive effort can also be allocated to processing and experiencing other mental content. Based on this line of reasoning, our main prediction is that the relationship between text difficulty and mind wandering should be U-shaped. That is, we predict that people should be most likely to mind wander when they are reading (very) easy and (very) difficult texts, compared to when they are reading texts that are moderately difficult.

In addition, we assume that people assess the potential benefits of reading a text as higher when they experience a text as more interesting. As potential benefits can compensate for potential costs, we further predicted that the U-shaped effect of text difficulty should be less pronounced (i.e., flatter) when people experience the text as more interesting.

## The present research

Going beyond prior work (see Feng et al. 2013; Forrin et al. 2019; Mills et al. 2013, 2015), we designed an experiment in which participants were exposed to texts of five—instead of only two—difficulty levels. We collected reading materials that naturally vary in difficulty on a continuum, rather than using a binary manipulation that enabled us to test the hypothesis that the relation between text difficulty and mind wandering is U-shaped. As in previous work, participants were sometimes interrupted by probes. In response to these probes, participants indicated whether they were mind wandering.

In making our design choices, we prioritized high ecological validity. Therefore, we selected non-fiction texts about themes that could well feature in college-level courses. Rather than modifying these texts to be more or less difficult (as prior work did), we carefully selected texts from similar sources, about similar topics, but that varied in difficulty. To mirror natural reading, we presented the texts page by page instead of sentence by sentence.

We also examined the role of interest in the text as a motivational factor. Previously, interest was found to predict mind wandering regardless of text difficulty (Fulmer et al. 2015; Unsworth and McMillan 2013). Furthermore, some studies suggested that interest mediates the relationship between text difficulty and mind wandering (Giambra and Grodsky 1989; Soemer and Schiefele 2019). In the current study, we reasoned as follows: if (a) the allocation of cognitive control results from cost–benefit analysis, and if (b) people’s interest in a text inputs in such cost–benefit analysis, the effect of text difficulty should be suppressed when people find a text more interesting. After all, when they read less interesting texts, people should allocate less control effort to reading regardless of difficulty, thus leaving more room to process unrelated mental content (see Fulmer et al. 2015).

Finally, we aimed to replicate the well-established finding that mind wandering is associated with decrements in reading comprehension (Feng et al. 2013; Mrazek et al. 2013; Soemer and Schiefele 2019) using our newly developed stimulus materials.

## Methods

### Participants and design

We originally planned to recruit 80 participants, as power simulations suggested that 80 participants would be sufficient to detect an effect size of OR 1.24 (from Feng et al. 2013) with ~ 80% power. However, 17 additional people expressed interest in participating, and we decided to allow them. Thus, 97 participants completed the study (*M*_age_ = 22.4, SD = 2.9; 76 women, 21 men), all university students. Of these participants, we excluded 7 before data analyses using pre-registered criteria (no variance in self-reported mind wandering, 4; no variance in perceived text difficulty, 2; age over 30, 1). Since earlier research has shown that the prevalence of mind wandering differs between age groups (Jordaõ et al. 2019), and since we aimed to recruit a homogenous sample of university students, we decided to include participants between 18 and 30 years of age. For one additional participant, no data were stored due to a software error. Thus, data from 89 participants were included in our analyses.

The main independent variable in the experiment was text difficulty, which we manipulated within-subjects. As an additional independent variable, we measured text interest after each text. The main dependent variable was the occurrence of mind wandering during reading. We pre-registered our planned sample size, exclusion criteria, hypotheses, and analysis plan on https://aspredicted.org/bk6a6.pdf.

### Materials

We used ten text passages. We gathered these text passages by searching in popular-science magazines and scientific journals with different target groups (children, interested lay audience, college students, academics). Specifically, we searched for articles about either of two topics, *animals* and *politics*, using search terms, such as *animals*, *politics*, *bees*, *penguins*, *cold war*, and *civil war*. We found 67 articles that were potentially suitable (see https://osf.io/s8ery/).

We proceeded by selecting the easiest and the most difficult passages, using a computer algorithm that computes Flesch–Kincaid Grade Levels (FKGL). This algorithm calculates words-per-sentence ratio and syllables-per-words ratio to construct a score that reflects the US school grade level. After selecting very easy (level 1) and very difficult (level 5) texts, we selected texts with moderate difficulty levels (levels 2–4). We ensured that the steps between levels were approximately equal, based on the FKGLs. In total, we selected five texts with varying difficulty levels per topic (see Table 1).

**Table 1:** Characteristics of the stimulus materials

Topic	Word count	Difficulty level	FKGL	Readability	Simplicity (%)	Narrativity (%)	Referential cohesion (%)	Deep cohesion (%)	Formality	M Interest	M perceived difficulty
Animals	742	1	5.8	13.6	83.9	52.8	31.9	52.8	− 0.46	3.4	1.9
Animals	1005	2	8.3	9.9	60.6	23.0	15.4	55.6	− 0.08	3.4	2.3
Animals	848	3	11.9	7.9	22.7	30.5	40.9	35.9	0.06	3.0	2.3
Animals	1193	4	13.4	7.3	20.9	22.7	52.8	57.1	− 0.28	3.0	2.3
Animals	849	5	15.7	8.5	34.5	20.6	71.2	68.1	0.30	2.0	3.2
History	939	1	7.1	16.7	92.9	39.4	16.9	78.5	− 0.52	3.8	1.9
History	1088	2	9.1	15.7	25.5	45.6	26.8	36.3	0.24	2.8	3.0
History	889	3	11.1	14.4	63.3	33.0	16.6	83.9	− 0.09	3.4	2.6
History	1063	4	13.6	12.7	57.9	39.0	9.3	92.5	0.01	2.3	2.8
History	1482	5	16.1	4.1	44.0	11.9	3.3	42.5	0.02	2.4	2.9

Before finalizing our selection, we used the Coh–Metrix online tool (Graesser et al. 2011; Graesser et al. 2014) to do a multi-dimensional text analysis, providing a deeper examination of difficulty levels for all texts. This was done to verify the difficulty rankings that we established using FKGL. The Coh–Metrix scores (Table 1, columns 6–11) generally confirmed that the selected texts could be characterized by five increasing difficulty levels with approximately equal steps between levels.

### Procedure

Upon arrival, participants were seated in a cubicle. A computer script presented all stimuli and recorded all measurements. This script first gave participants a definition of mind wandering based on previous studies (Feng et al. 2013; Smallwood and Schooler 2006): “Mind wandering describes a state of mind that occurs when your attention shifts away from the task that you are doing at that moment”. Participants were instructed to read text passages at their own pace; they learned that they would spend 3 min on each passage (regardless of their pace).

Texts appeared in random order. Each text consisted of several pages, with roughly the same number of words on page one and two, and the variation in the length of text showing on page 3 (page 1: *M* = 399, SD = 22; page 2: *M* = 414, SD = 56; page 3: *M* = 199, SD = 141; average: *M* = 380, SD = 64). Participants could flip to the next page by pressing the spacebar; they could not go back to the previous page. A pilot test, in which participants (*N* = 8) freely read all texts, showed that the fastest readers finished the shortest texts in ± 3 min. Based on this, we set the time restriction mentioned earlier to 3 min, to ensure that participants would not mind wander because they had finished reading.

Within each reading period, participants were interrupted with one thought probe asking, “Were you just mind wandering?” with the answer options *yes* and *no*. The probes were presented at a random moment between 1.0 and 2.5 min after the onset of each reading interval. After the probe, participants continued reading until the 3-min period was over. After each reading period, participants answered two items about interest (How interesting did you find this text? 1 = not interesting at all; 5 = very interesting) and perceived text difficulty (How difficult did you find this text? 1 = very easy; 5 = very difficult). Finally, after reading all texts, participants completed a comprehension test. This test consisted of 30 multiple-choice questions, three per article, each question involving four answer options. The questions were designed to test the recollection of the explicit information from the respective text. All questions were about the content of the first page of each text, so that even slow readers would, in principle, still be able to correctly answer the questions.

### Analyses

We used generalized mixed-effects models to analyze our data, using the *lme4* package in R (Bates et al. 2014). In all models, unless otherwise mentioned, mind wandering (*yes* vs. *no*, binary) was the dependent variable. All models included a fixed intercept, and two main effects of text difficulty, one linear and one quadratic. The main effects were both treated as continuous variables. To take into account that some people may generally mind wander more than others, we included per-participant adjustments to the intercept (i.e., a random intercept) in all models. Following well-established guidelines (Barr et al. 2013), we also included per-participant adjustments to the linear and quadratic effects of text difficulty (i.e., random slopes). These random slopes account for the possibility that some people are more responsive to the difficulty manipulation than others. Moreover, to take into account that either of the text topics may generally be more conductive to mind wandering, a per-topic adjustment to the intercept (i.e., another random intercept) was included. Finally, models included all correlations among the random effects. All continuous predictors were centered around the sample mean.

In all models, some of the variances were estimated to be zero or very close to zero (i.e., we encountered a ‘singularity warning’). Thus, as sensitivity analyses, we reproduced all our main analyses with models that had a simplified random-effects structure, i.e., models that included only per-participant adjustments to the intercept, and no other random effects. A report of these sensitivity analyses is included in the Supplementary Information (see Appendix, Table S1). These analyses yielded similar estimates as our main analyses, and are thus not discussed further.

​To replicate the previous finding that mind wandering harms text comprehension (e.g. Feng et al. 2013; Mrazek et al. 2013), we used a generalized mixed-effects model, akin to the models we used to test our main hypotheses but with comprehension as the dependent variable.

## Results

Unless otherwise noted, all analyses reported below were pre-registered.

### Descriptive statistics

Participants reported that they were mind wandering at 38.9% of the thought probes. Most of the thought probes (74%) appeared while participants were reading the first page of a text (page 2: 23%; page 3: 2%) which suggests that on average participants read slower than we expected beforehand. The overall proportions of mind wandering per person were similar depending on which text difficulty level participants read first (difficulty level one: *M* = 0.42, SD = 0.19; two: *M* = 0.36, SD = 0.18; three: *M* = 0.33, SD = 0.22; four: *M* = 0.43, SD = 0.19; five: *M* = 0.37, SD = 0.21). On average, participants perceived the texts as moderately interesting (*M* = 2.9, SD = 1.3) and moderately difficult (*M* = 2.5, SD = 1.1). Table 1 (column 12 and 13) includes the average ratings on perceived text difficulty and text interest per objective text difficulty level.

The correlation between perceived difficulty and the objective difficulty of the texts was significant (*r* = 0.28, *p* < 0.001), suggesting that the difficulty manipulation was successful. Mind wandering occurrences were mildly positively related to difficulty and perceived difficulty, suggesting that people’s minds wandered more the more difficult a text was and the more difficult it was perceived to be (see Table 2). Furthermore, Table 2 shows that text interest was negatively related to the other three variables. People tended to be less interested in texts that were more difficulty or perceived to be more difficult. Also, people reported less mind wandering for texts they found more interesting.

**Table 2:** Correlations between mind wandering, difficulty, perceived difficulty and interest

	1	2	3
1. Mind Wandering	–		
2. Difficulty	0.16*	–	
3. Perceived Difficulty	0.13*	0.28*	–
4. Interest	− 0.39*	− 0.36*	− 0.28*

These correlations are on the level of the individual texts; they neglect the nested structure of our data (i.e., texts are nested within participants). *N* = 890; *p < 0.001

During the comprehension test, participants correctly answered 61% (SD = 11) of the questions. Accuracy was somewhat lower for questions on the texts about animals (*M* = 54%, SD = 15) than the texts about history (*M* = 67%, SD = 13). Moreover, participants’ performance on the questions about history generally decreased with text difficulty (difficulty level one: *M* = 82%, two:* M* = 64%, three: *M* = 76%; four: *M* = 63%; five: *M* = 48%), whereas we could not recognize this pattern for the texts on animals (difficulty level one: *M* = 40%, two:* M* = 69%, three: *M* = 51%; four: *M* = 44%; five: *M* = 67%).

### Does text difficulty predict mind wandering?

**Fig. 1:**
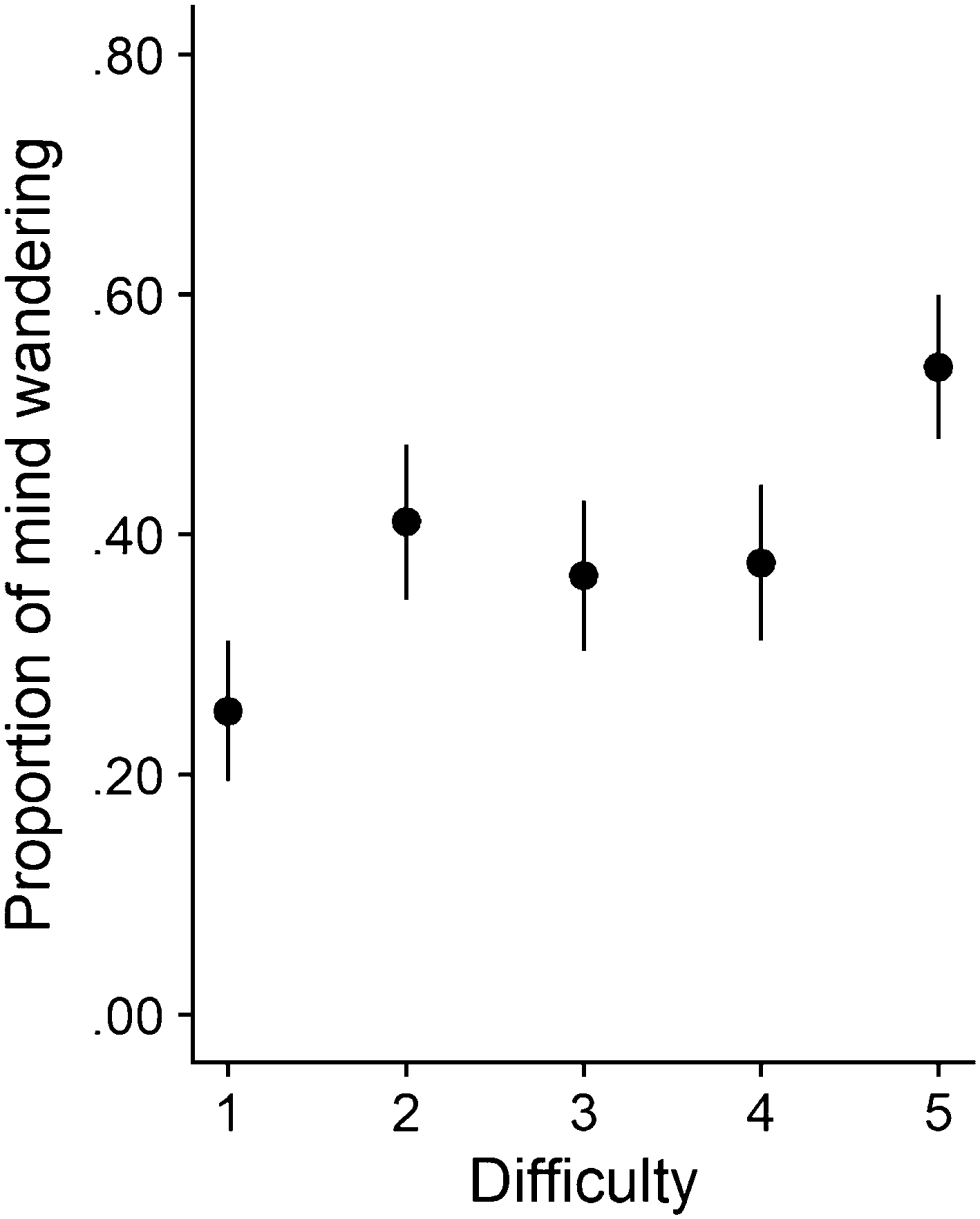
Mean proportion of mind wandering as a function of text difficulty. Error bars reflect within-subjects 95% confidence intervals (Cousineau, 2005)

As described above, we ran a generalized mixed-effects model to examine the effect of text difficulty on mind wandering (Table 3, Model 1). Results indicated no significant effect for the quadratic predictor of text difficulty on mind wandering (OR 1.02, *p* = 0.698). However, we found a significant linear effect of text difficulty on mind wandering (OR 1.29, *p* < 0.001). With increasing text difficulty, participants' minds wandered more (Fig. 1). Although findings do not reveal the hypothesized quadratic pattern, they are consistent with prior work showing that people’s mind wanders more while reading more difficult texts (Feng et al. 2013; Mills et al. 2013).

**Table 3:** Overview of results from mixed-level linear models

Model	Term	Est	95% CI	Z	p	OR
1	Intercept	− 0.51	[− 0.70, − 0.32]	− 5.20	<0 .001	0.60
	Text difficulty (linear)	0.25	[0.14, 0.36]	4.51	< 0.001	1.29
	Text difficulty (quadratic)	0.02	[− 0.07, 0.11]	0.39	0.698	1.02
2	Intercept	− 0.66	[− 0.88, − 0.43]	− 5.71	< 0.001	0.52
	Text difficulty (linear)	− 0.00	[− 0.13, 0.12]	− 0.06	0.956	1.00
	Text difficulty (quadratic)	− 0.03	[− 0.13, 0.07]	− 0.56	0.576	0.97
	Text interest	− 0.85	[− 1.05, − 0.65]	− 8.19	< 0.001	0.43
	Text difficulty (quadratic) × text interest	− 0.07	[− 0.15, 0.02]	− 1.57	0.117	0.93
3	Intercept	− 0.61	[− 0.86, − 0.36]	− 4.78	0.000	0.54
	Text difficulty (linear)	0.07	[− 0.08, 0.21]	0.93	0.354	1.07
	Text difficulty (quadratic)	0.02	[− 0.09, 0.13]	0.41	0.684	1.02
	Text interest	− 0.89	[− 1.11, − 0.67]	− 7.91	< 0.001	0.41
	Text difficulty (linear) × text interest	0.15	[0.02, 0.29]	2.23	0.026	1.16

### Does interest play a role in mind wandering?

To examine whether the effect of text difficulty is moderated by text interest, we extended the model described in the previous paragraph with (a) the fixed main effect of text interest and (b) the fixed interaction between text interest and text difficulty (quadratic). We also added the corresponding random slopes. Results appear in Table 3 (Model 2). In short, text difficulty did not predict mind wandering in this model, neither as a linear nor as a quadratic predictor. The main effect of text interest, however, was significant (OR 0.43*, p* < 0.001) indicating that the more interested participants were in the text, the less their mind wandered. Contrary to our prediction, the interaction between interest in the text and the quadratic predictor of text difficulty was not significant (OR 0.93, *p* = 0.117).

In Model 1 (see Table 3), we did find a clear linear effect of text difficulty on mind wandering which we originally did not expect. Thus, we explored the possibility that this linear effect was moderated by text interest in Model 3. In this analysis (see Table 3), which we had not pre-registered, we indeed found an interaction between the linear predictor of text difficulty and text interest (OR 1.16, *p* = 0.026).^1^

**Fig. 2:**
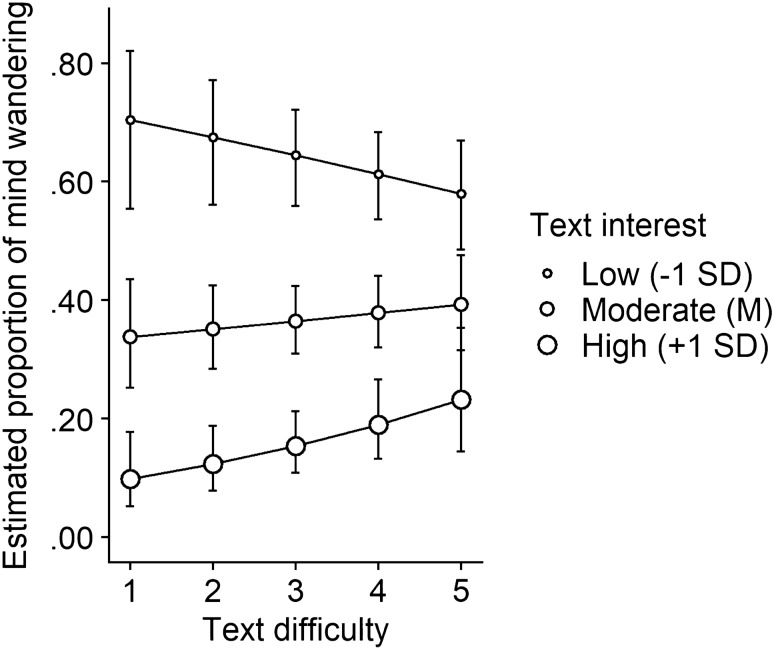
Estimated proportions of mind wandering, as a function of text difficulty and text interest. Estimates are based on Model 3 (see Table 3 and main text). Error bars indicate 95% confidence intervals

To interpret this interaction, we calculated the simple slope estimates derived from Model 3 with the *interactions* package in R (Long 2019) and plotted these estimates in Fig. 2. Inspection of Fig. 2 suggests that the linear effect of difficulty on mind wandering (more mind wandering with difficult texts) shifted depending on whether people found the text interesting. When people found a text uninteresting (–1 SD), mind wandering did not substantially differ depending on text difficulty (Est = − 0.14, SE = 0.10, *p* = 0.176, OR = 0.87). When people perceived the text as somewhat interesting (i.e., at average interest level), mind wandering also did not substantially differ depending on text difficulty (*Est* = 0.05, SE = 0.07, *p* = 0.411, OR = 1.06). Only when people thought a text was very interesting did their minds wander more during more difficult texts (Est = 0.26, SE = 0.13, *p* = 0.043, OR = 1.29). In sum, while there was an interaction between text difficulty and text interest, this interaction was different from our expectations. That is, the previously found positive relation between difficulty and mind wandering was clearest when text interest was high (+ 1SD). We return to this issue in “Discussion” section.

### Does mind wandering impair text comprehension?

To replicate prior findings, we tested whether mind wandering predicted text comprehension. To this end, we conducted a linear mixed-effects model with text comprehension (i.e. correctly answered questions per text) as dependent variable and mind wandering as a fixed effect. Taking into account that some people could generally comprehend more than others, we also included per-participant adjustments to the intercept of text comprehension (i.e., a random intercept). As predicted, we found that participants who mind wandered during a text performed worse on the respective text comprehension questions (Est. = − 0.06, SE = 0.02, *p* = 0.003).^2^

Next, though we had no specific hypotheses about this, we explored whether the association between mind wandering and text comprehension was part of a longer causal chain. To this end, we conducted mediation analyses, using a procedure suitable for assessing mediation in mixed-level models (texts nested within participants; Imai et al. 2010). We used this procedure to decompose the total effect of difficulty on text comprehension into a direct effect (difficulty → text comprehension) and an indirect effect (difficulty → mind wandering → text comprehension). This analysis revealed that there was a significant direct effect of difficulty on text comprehension, Est =  − 0.22, 95% CI [− 0.38, − 0.06], *p* = 0.006, which explained 88.5% of the total effect, but also an indirect effect via mind wandering, Est =  − 0.03, 95%CI [− 0.06, − 0.004], *p* = 0.021, which explained 11.5% of the total effect. Thus, the results are consistent with the notion that difficulty affects text comprehension in part through its effect on mind wandering.

## Discussion

We found mind wandering increased linearly as people read more difficult texts. This finding extends previous work in two ways. First, our experiment suggests that a linear relationship between difficulty and mind wandering exists during natural, page-by-page reading. Second, our study suggests that this relationship holds across a large range of difficulty levels (i.e., 9 US grade levels). Although these findings replicate and extend previous work (Feng et al. 2013; Mills et al. 2013; Soemer et al. 2019; Soemer and Schiefele 2019), they do not support our novel hypothesis that mind wandering is lowest at moderate levels of difficulty, and highest at very easy and very difficult levels, even though our study was designed to be able to detect nonlinear effects.

Post hoc, our findings can still be reconciled with cost–benefit models of cognitive effort (Shenhav et al. 2017), when we only assume that (a) building a situational model requires cognitive effort, and (b) people may sometimes decide that the costs of such effort are not, or not anymore, worth expending. In our view, these two assumptions are plausible; at least, they are consistent with our findings*,* with modern theories of mind wandering (that suggest that at least some mind wandering is voluntary; Seli et al. 2016a, b), and with modern theories of cognitive control (that suggest that the allocation of control can be understood as resulting from a cost–benefit decision-making process; Shenhav et al. 2017). Thus, we think these assumptions can be used to generate further hypotheses regarding the interaction of task parameters (e.g., related to text difficulty) and motivational states (e.g., related to teacher expectations in educational settings) affecting mind wandering. However, we also reasoned a priori that when people allocate less cognitive effort to reading (i.e., when they are reading easy texts), they are more likely to mind wander. This idea seems incompatible with our findings.

Exploratory analyses suggested that the effect of difficulty was most pronounced for texts that people found very interesting; we found no evidence for an effect of difficulty for texts that people found uninteresting. At first sight, the direction of this interaction seems at odds with the one reported by Fulmer et al. (2015), who found that the effect of difficulty was most pronounced for texts that people found uninteresting; for interesting texts, people’s mind wandered little regardless of difficulty. We should note, though, that the study by Fulmer et al. (2015) was methodologically different from ours. In that study, the difficulty range of the texts was much smaller (i.e., 2 US grade levels), and the method used to manipulate interest was based on readers’ expectations before reading, not actual experiences. So, there may be several reasons for why the findings were not more similar. Still, both studies together suggest that, when trying to examine difficulty effects on mind wandering, it is wise to take reading motivation and interest into account. This conclusion also aligns with Soemer and Schiefele’s (2019) finding that the effect of text interested fully mediated the relation between text difficulty and mind wandering.

The finding that difficulty affects mind wandering especially for texts that people perceive as interesting can be explained post-hoc by positing that people are categorically unwilling to invest substantial cognitive effort into building a situational model when they are not interested in the text; thus, the effect of difficulty becomes visible only when people are interested in the text. We note, though, that the test of this text difficulty * text interest interaction (a) was not pre-registered and (b) failed the sensitivity analysis in which we substituted objective (manipulated) difficulty with perceived difficulty (see Footnote 1). Even though this interaction is intriguing, it should be interpreted with great caution.

We replicated the ubiquitous finding that mind wandering impairs reading comprehension (e.g. Mrazek et al. 2013). Beyond this replication, exploratory analysis suggested the existence of an indirect route to failures of text comprehension: people fail to comprehend difficult texts not only because these texts are difficult per se, but also because these texts are more conductive to mind wandering. The existence of this indirect pathway suggests that the maximum level of text difficulty people can comprehend is not just constrained by people’s reading ability, but also by their mind’s tendency to drift off.

Our results show no support for the hypothesis that mind wandering is related to text difficulty in a U-shaped manner. Specifically, we found no evidence of people’s minds wandering more while they read (very) easy texts. Speculatively, we may have underestimated the cognitive effort it takes to build a situational model while reading, even for easy texts. That is, people may need to allocate cognitive effort mainly on reading to comprehend a text, even if that text is very easy. Further research—that employs behavioral, physiological, or subjective measures of effort (Bijleveld 2018; Scheiter et al. 2020)—may help to better understand whether and how the effort costs of building situational models can explain mind wandering during reading.

Another open question that pertains regarding the relation between text difficulty and mind wandering is whether such difficulty-triggered mind wandering is unintentional or intentional. Prior studies suggest both are possible (Seli et al. 2016a, b; Soemer and Schiefele 2019), but the mechanisms through which intentional vs. unintentional mind wandering are triggered still need to be explored further. A promising avenue is to look at this issue through the lens of models of motivation–cognition interactions, like we did in the present study. Speculatively, both types of mind wandering may be underpinned by different cost–benefit weighting mechanisms (e.g., different in that they do vs. do not involve conscious awareness; Zedelius et al. 2014). Future research is needed to test this possibility.

In the present study, we used text interest as the main motivational factor when exploring the possible meaning of the models of motivation–cognition interactions for mind wandering. Time-on-task might be another relevant motivational factor to take into account, however. We controlled for this factor at the group level by randomly assigning the sequence of texts among the participants. However, it is important to note that previous studies have shown that time-on-task is an important motivational predictor of mind wandering on its own. Specifically, studies that measured mind wandering during cognitive tasks (e.g. working memory tasks) have shown that mind wandering increases with increased time-on-task (Brosowsky et al. 2020; Krimsky et al. 2017; Thomson et al. 2014). To explain this finding, Browosky et al. (2020) suggested that at the start of a cognitive task—which is often new to participants—people allocate relatively high on-task focus, since they are then still lacking sufficient knowledge on the task’s costs and benefits (see also Kurzban et al. 2013). With time, they may sometimes learn that high performance has no benefits other than helping the researcher, after which they disengage However, when reading, participants may still see the personal benefit of learning something new. Future studies have yet to identify how the findings regarding other cognitive tasks, each of which has their unique costs and benefits, translate into the context of mind wandering while reading.

While the current study could support earlier findings and provide new perspectives, it also has some limitations. One limitation to the ecological validity is that we limited the time participants had to read each text and that they could not go back to earlier pages. We made this choice to ensure that participants can finish the task within the planned time, and to ensure that the effect of mind wandering on text comprehension cannot be biased by participants who choose to re-read passages.

Another limitation of our study is that we did not experimentally manipulate the text difficulties of the texts but chose texts based on their FKGL scores. Thus, it is possible that other text characteristics besides text difficulty influenced the current results. Previous studies did experimentally manipulate text difficulty by changing the words or sentence structure of a text without changing the content (Feng et al. 2013; Fulmer et al. 2015; Mills et al. 2013; Soemer et al. 2019; Soemer and Schiefele 2019). While this is a more controlled manipulation, we chose against it, as it would not allow the range in text difficulty that we strived for in the current study.

Related to the previous point, we primarily used FKGL to categorize our texts in text difficulty. FKGL is an often-used measure of text difficulty. However, it is also often criticized for its simplicity as its formula only relies on letters-per-word and words-per-sentence ratios (Dufty et al. 2006; Forrin et al. 2019; Fulmer et al. 2015; Graesser et al. 2011). Forrin et al.’ experiments (2019) showed that the effects of text difficulty measured with the FKGL on mind wandering could largely be explained through an effect of section length rather than text difficulty itself. Coh–Metrix takes more facets of text difficulty into account by providing several measures of text difficulty. While we took the Coh–Metrix metrics (Graesser et al. 2011) into account while choosing our materials, it is hard, perhaps impossible, to find texts that present exactly the same pattern of text difficulty in all Coh–Metrix measures, or even on a composite Coh–Metrix measure (i.e., ‘Formality’; Graesser et al., 2014). In other words, we acknowledge that text difficulty is not a unitary construct, even though we did treat it as such in the present study.

In sum, the current study showed that people’s minds tend to wander more with increasing text difficulty and decreased interest. Mind wandering in turn explains at least in part why more difficult texts lead to lower reading comprehension. From an applied perspective, our findings highlight the merits of the classic advice for writers to simplify their writing (“avoid fancy words”, “use the active voice”, “avoid the use of qualifiers”, “omit needless words”; Strunk and White 1959). Heeding such advice may well help readers mind wander less.

## Supplementary Information

Below is the link to the electronic supplementary material.

Supplementary file1 (DOCX 16 KB)

## Data Availability

Materials, data, and scripts can be downloaded from https://osf.io/s8ery/.

## Footnotes

1 We thank one reviewer for the suggestion to conduct sensitivity analyses on the our main analyses using the linear and quadratic effect of perceived text difficulty instead of objective text difficulty. The outcomes for Model 1 and Model 2 were similar to the outcomes described above; however, we could not replicate the interaction effect found in Model 3 (see Table S2).

2 Our analysis on the relation between mind wandering and text comprehension was to some degree mismatched since we only measured participants’ text comprehension of the first page, while we measured mind wandering independent of the page, i.e., probes could occur at all times. To explore whether this mismatch affected the outcome of our analysis, we added a main effect of the page number (i.e. the page participants were reading while probed for mind wandering) and an interaction effect of page number*mind wandering to our model. The magnitude of the relation between mind wandering remained approximately the same (Est. = − 0.07, SE = 0.02, *p* = 0.004) and we found no evidence that the page number moderated the relation between mind wandering and text comprehension (Est. = 0.01, SE = 0.02, *p* = 0.533).

## References

[CR1] Aridan N, Malecek NJ, Poldrack RA, Schonberg T (2019). Neural correlates of effort-based valuation with prospective choices. Neuroimage.

[CR2] Barr DJ, Levy R, Scheepers C, Tily HJ (2013). Random effects structure for confirmatory hypothesis testing: Keep it maximal. Journal of Memory and Language.

[CR3] Bates, D., Mächler, M., Bolker, B., & Walker, S. (2014). *Fitting linear mixed-effects models using lme4*. http://arxiv.org/abs/1406.5823.

[CR4] Bijleveld E (2018). The feeling of effort during mental activity. Consciousness and Cognition.

[CR5] Broadway JM, Franklin MS, Schooler JW (2015). Early event-related brain potentials and hemispheric asymmetries reveal mind-wandering while reading and predict comprehension. Biological Psychology.

[CR6] Brosowsky NP, DeGutis J, Esterman M, Smilek D, Seli P (2020). Mind wandering, motivation, and task performance over time: Evidence that motivation insulates people from the negative effects of mind wandering. Theory, Research, and Practice Psychology of Consciousness.

[CR7] Cousineau D (2005). Confidence intervals in within-subject designs: A simpler solution to Loftus and Masson’s method. Tutorial in Quantitative Methods for Psychology.

[CR8] Dora, J., van Hooff, M., Geurts, S., Kompier, M., & Bijleveld, E. (2020). Labor/leisure decisions in their natural context: The case of the smartphone. *Psychonomic Bulletin & Review*, 1-10. 10.3758/s13423-020-01844-2 10.3758/s13423-020-01844-2 PMC8062367 33219457

[CR9] Dufty, D. F., Graesser, A. C., Louwerse, M. M., & Mcnamara, D. S. (2006). Assigning grade levels to textbooks: Is it just readability? *The 28th annual conference of the cognitive science society*, 1251–1256.

[CR10] Dunn TL, Lutes DJ, Risko EF (2016). Metacognitive evaluation in the avoidance of demand. Journal of Experimental Psychology: Human Perception and Performance.

[CR11] Feng S, D’Mello S, Graesser AC (2013). Mind wandering while reading easy and difficult texts. Psychonomic Bulletin and Review.

[CR12] Forrin ND, Risko EF, Smilek D (2019). On the relation between reading difficulty and mind-wandering: A section-length account. Psychological Research Psychologische Forschung.

[CR13] Fulmer SM, D’Mello SK, Strain A, Graesser AC (2015). Interest-based text preference moderates the effect of text difficulty on engagement and learning. Contemporary Educational Psychology.

[CR14] Giambra LM, Grodsky A, Shorr JE, Robin P, Connella JA, Wolpin M (1989). Task-unrelated images and thoughts while reading. Imagery.

[CR15] Graesser AC, McNamara DS, Cai Z, Conley M, Li H, Pennebaker J (2014). Coh–Metrix measures text characteristics at multiple levels of language and discourse. The Elementary School Journal.

[CR16] Graesser AC, McNamara DS, Kulikowich JM (2011). Coh–Metrix: Providing multilevel analyses of text characteristics. Educational Researcher.

[CR17] Hull, C. L. (1943). Principles of behavior. New York: Appleton-century-crofts

[CR18] Imai K, Keele L, Tingley D (2010). A general approach to causal mediation analysis. Psychological Methods.

[CR19] Inzlicht M, Shenhav A, Olivola CY (2018). The effort paradox: Effort is both costly and valued. Trends in Cognitive Sciences.

[CR20] Jordaõ M, Ferreira-Santos F, Pinho MS, St Jacques PL (2019). Meta-analysis of aging effects in mind wandering: Methodological and sociodemographic factors. Psychology and Aging.

[CR21] Kane MJ, McVay JC (2012). What mind wandering reveals about executive-control abilities and failures. Current Directions in Psychological Science.

[CR22] Kool W, McGuire JT, Rosen ZB, Botvinick MM (2010). Decision making and the avoidance of cognitive demand. Journal of Experimental Psychology: General.

[CR23] Krimsky, M., Forster, D. E., Llabre, M. M., & Jha, A. P. (2017). The influence of time on task on mind wandering and visual working memory. *Cognition*, *169*, 84-90. 10.1016/j.cognition.2017.08.006 10.1016/j.cognition.2017.08.006 28865286

[CR24] Kurzban R, Duckworth A, Kable JW, Myers J (2013). An opportunity cost model of subjective effort and task performance. Behavioral and brain sciences.

[CR25] Lindquist SI, McLean JP (2011). Daydreaming and its correlates in an educational environment. Learning and Individual Differences.

[CR26] Long, J.A. (2019). *Interactions: Comprehensive, user-friendly toolkit for probing interactions* [R package version 1.1.0]. https://cran.r-project.org/package=interactions.

[CR27] Mills C, D’Mello S, Lehman B, Bosch N, Strain A, Graesser A, Lane HC, Yacef K, Mostow J, Pavlik P (2013). What makes learning fun? Exploring the influence of choice and difficulty on mind wandering and engagement during learning. Artificial intelligence in education.

[CR28] Mills C, Dmello SK, Kopp K (2015). The influence of consequence value and text difficulty on affect, attention, and learning while reading instructional texts. Learning and Instruction.

[CR29] Mrazek MD, Phillips DT, Franklin MS, Broadway JM, Schooler JW (2013). Young and restless: Validation of the Mind-Wandering Questionnaire (MWQ) reveals disruptive impact of mind-wandering for youth. Frontiers in Psychology.

[CR30] Saunders B, Lin H, Milyavskaya M, Inzlicht M (2017). The emotive nature of conflict monitoring in the medial prefrontal cortex. International Journal of Psychophysiology.

[CR31] Scheiter K, Ackerman R, Hoogerheide V (2020). Looking at mental effort appraisals through a metacognitive lens: Are they biased?. Educational Psychology Review.

[CR32] Seli P, Cheyne JA, Xu M, Purdon C, Smilek D (2015). Motivation, intentionality, and mind wandering: Implications for assessments of task-unrelated thought. Journal of Experimental Psychology: Learning, Memory, and Cognition.

[CR33] Seli P, Risko EF, Smilek D (2016). On the necessity of distinguishing between unintentional and intentional mind wandering. Psychological Science.

[CR34] Seli P, Risko EF, Smilek D, Schacter DL (2016). Mind-wandering with and without intention. Trends in Cognitive Sciences.

[CR35] Seli P, Schacter DL, Risko EF, Smilek D (2019). Increasing participant motivation reduces rates of intentional and unintentional mind wandering. Psychological Research Psychologische Forschung.

[CR36] Shenhav A, Musslick S, Lieder F, Kool W, Griffiths TL, Cohen JD, Botvinick MM (2017). Toward a rational and mechanistic account of mental effort. Annual Review of Neuroscience.

[CR37] Smallwood J (2010). Why the global availability of mind wandering necessitates resource competition: Reply to McVay and Kane (2010). Psychological Bulletin.

[CR38] Smallwood J (2011). Mind-wandering while reading: Attentional decoupling, mindless reading and the cascade model of inattention. Linguistics and Language Compass.

[CR39] Smallwood J, McSpadden M, Schooler JW (2008). When attention matters: The curious incident of the wandering mind. Memory and Cognition.

[CR40] Smallwood J, Schooler JW (2006). The restless mind. Psychological Bulletin.

[CR41] Smallwood J, Schooler JW (2015). The science of mind wandering: Empirically navigating the stream of consciousness. Annual Review of Psychology.

[CR42] Soemer A, Idsardi HM, Minnaert A, Schiefele U (2019). Mind wandering and reading comprehension in secondary school children. Learning and Individual Differences.

[CR43] Soemer A, Schiefele U (2019). Text difficulty, topic interest, and mind wandering during reading. Learning and Instruction.

[CR44] Strunk W, White EB (1959). The elements of style.

[CR45] Thomson, D. R., Seli, P., Besner, D., & Smilek, D. (2014). On the link between mind wandering and task performance over time. *Consciousness and cognition, 27,* 14-26. 10.1016/j.concog.2014.04.001 10.1016/j.concog.2014.04.001 24780348

[CR46] Unsworth N, McMillan BD (2013). Mind wandering and reading comprehension: Examining the roles of working memory capacity, interest, motivation, and topic experience. Journal of Experimental Psychology: Learning Memory and Cognition.

[CR47] Zedelius CM, Veling H, Custers R, Bijleveld E, Chiew KS, Aarts H (2014). A new perspective on human reward research: How consciously and unconsciously perceived reward information influences performance. Cognitive, Affective, and Behavioral Neuroscience.

