# Supplementary material for: Mind wandering increases linearly with text difficulty
Source: Psychol Res. 2021 Feb 12;86(1):284–93. doi: 10.1007/s00426-021-01483-9 (PMC8821482; doi:10.1007/s00426-021-01483-9)
Supplement: Supplementary file 1 — Supplementary file1 (DOCX 16 KB) [file 426_2021_1483_MOESM1_ESM.docx]

**Appendix – Supplementary information**

| **Model** | **Term** | **Est.** | **95% CI** | **Z** | **p-value** | **OR** |
| --- | --- | --- | --- | --- | --- | --- |
| 1 | Intercept | -0.50 | [-0.69, -0.31] | -5.24 | <.001 | 0.61 |
|  | Text difficulty (linear) | 0.25 | [0.15, 0.35] | 4.77 | <.001 | 1.28 |
|  | Text difficulty (quadratic) | 0.02 | [-0.07, 0.10] | 0.38 | .702 | 1.02 |
| 2 | Intercept | -0.64 | [-0.85, -0.42] | -5.83 | <.001 | 0.53 |
|  | Text difficulty (linear) | 0.01 | [-0.12, 0.13] | 0.10 | 0.923 | 1.01 |
|  | Text difficulty (quadratic) | -0.02 | [-0.11, 0.08] | -0.35 | 0.724 | 0.98 |
|  | Text interest | -0.77 | [-0.92, -0.62] | -9.86 | <.001 | 0.46 |
|  | Text difficulty (quadratic) × Text interest | -0.06 | [-0.15, 0.02] | -1.49 | 0.135 | 0.94 |
| 3 | Intercept | -0.58 | [-0.80, -0.36] | -5.21 | <.001 | 0.56 |
|  | Text difficulty (linear) | 0.06 | [-0.06, 0.18] | 0.95 | 0.341 | 1.06 |
|  | Text difficulty (quadratic) | 0.03 | [-0.07, 0.12] | 0.50 | 0.617 | 1.02 |
|  | Text interest | -0.77 | [-0.92, -0.62] | -9.88 | <.001 | 0.46 |
|  | Text difficulty (linear) × Text interest | 0.10 | [0.00, 0.20] | 1.90 | 0.057 | 1.10 |

Table S1

*Results from mixed-level linear models with a simplified random-effects structure*

Table S2

*Results from mixed-level linear models with the linear and quadratic effect of perceived difficulty as independent variables*

| **Model** | **Term** | **Est.** | **95% CI** | **Z** | **p-value** | **OR** |
| --- | --- | --- | --- | --- | --- | --- |
| 1 | Intercept | -.48 | [-.72, -.25] | -4.00 | <.001 | 0.62 |
|  | Perceived text difficulty (linear) | .22 | [.03, .43] | 2.24 | .025 | 1.26 |
|  | Perceived text difficulty (quadratic) | -.09 | [-.22, 0.03] | -1.39 | .165 | .91 |
| 2 | Intercept | -.76 | [-1.00, -.42] | -4.79 | <.001 | .49 |
|  | Perceived text difficulty (linear) | .03 | [-.1, .24] | .24 | .807 | 1.03 |
|  | Perceived text difficulty (quadratic) | -.10 | [-.25, .05] | -1.27 | .206 | .91 |
|  | Text interest | -.88 | [-1.10, -.67] | -8.12 | <.001 | .41 |
|  | Perceived text difficulty (quadratic) × Text interest | -.07 | [-.17, .02] | -1.47 | .141 | .93 |
| 3 | Intercept | -.76 | [-1.12, -.40] | -4.17 | <.001 | .47 |
|  | Perceived text difficulty (linear) | -.02 | [-.30, .27] | -.11 | .915 | .98 |
|  | Perceived text difficulty (quadratic) | -.11 | [-.28, .06] | -1.28 | .199 | .89 |
|  | Text interest | -.85 | [-1.09, -.60] | -6.79 | <.001 | .43 |
|  | Perceived text difficulty (linear) × Text interest | .06 | [-.12, .23] | .66 | .512 | 1.06 |
